# Successful Liver Transplantation Case Report from a Deceased Donor with Sickle Cell Anemia

**DOI:** 10.1155/2018/5154136

**Published:** 2018-10-22

**Authors:** Lucas Souto Nacif, Estrella Bianca de Mello, Rafael Soares Pinheiro, Fabiana Roberto Lima, Rodrigo Bronze de Martino, Wellington Andraus, Luiz Carneiro D'Albuquerque

**Affiliations:** ^1^Liver and Gastrointestinal Transplant Division, Department of Gastroenterology, 05403-900 São Paulo, Brazil; ^2^Department of Pathology, University of São Paulo School of Medicine, 05403-900 São Paulo, Brazil

## Abstract

There is a worldwide problem of waiting time and mortality rate associated with remaining on the waiting list for a liver transplant. However, some situations have been encouraging in terms of determining appropriate recipients and expanding the donor criteria. We herein report a case of useful liver donor with sickle cell anemia for liver transplantation. Here we described a case of liver transplantation from a donor with sickle cell anemia to a recipient with hepatocellular carcinoma who was deemed to be at risk of tumor growth and at risk of being dropped from the waiting list. The literature reveals the importance of using safe donors, and we describe the benefits of using a safe, deceased liver donor with sickle cell anemia who was an adequate option for liver transplantation.

## 1. Introduction

Nowadays, due to the great success of liver transplantation (LT) given advances in technologies and the quality of immunosuppressive drugs, the survival and quality of life have improved [1, 2]. However, a major worldwide problem is still the waiting time and mortality rate associated with remaining on waiting lists [3, 4].

This situation has motivated revisions to the recipient-selection process and an expansion of donor criteria [5]. The risks and benefits of remaining on the waiting list for LT should always be evaluated closely when using doubtful grafts or adopting expanded criteria [1, 5]. Absolute contraindications to deceased organ donation include any evidence of a malignant cancer. Furthermore, other important liver grafts refusal was evaluated by histological analysis with severe steatosis, cholestasis, and fibrosis. Nevertheless, controversies in the relevance of these mandates require that the surgeon team decide the best way to allocate organs.

The purpose of this study is to show the possibility of enlarging use of organs. Moreover, we describe the special case report of a LT from a donor with sickle cell anemia (SCA) and hereditary hemoglobinopathy, with an atypical and inherited disorder of hemoglobin called hemoglobin S (HbS), to a hepatitis B virus (HBV) cirrhosis and hepatocellular carcinoma (HCC) patient who was adjudicated dropping out of the liver transplant waiting list.

## 2. Case Presentation

### 2.1. Donor Surgery

The liver graft was from a young male patient, 20 years old, who weighed 57 kg, was 1.78 meters tall, and had a body mass index (BMI) of 18 kg/m^2^. He was admitted to a general hospital with a subarachnoid hemorrhage and intracranial hematoma on the left side, and he suffered brain death. He had a previous pathological history of SCA and was treated for his anemia with several blood cell therapies and a splenectomy when he was 16 years old. He was receiving ceftriaxone, meropenem, and vancomycin when he was submitted to donor surgery, five days after the neurosurgery. He was under low doses of vasopressors: norepinephrine (0.18 mcg/kg/min) and vasopressin (0.02 mcg/kg/min). The best suitable recipient was chosen by balancing the risk of a hematological disease or thrombotic risk factors associated with the recipient remaining on the waiting list and either dying or dropping out of the list. All laboratory analysis and liver function of the donor were normal. After all analysis and arguments discussed with the transplant team (surgeons, hepatologists, and infectologists), as risk of using SCA graft, probabilities of developing disease, and a few case reports in the literature, on the other hand, the benefits of being an excellent hepatic graft option, the recipient and her family were informed of all risks and probabilities, and a unanimous informed consent decision was made to receive the donor liver and follow with the transplant. The donor surgery was fine and was not associated with any complications. Both of the deceased patient's kidneys and liver were donated to three different recipients in different centers.

### 2.2. Liver Transplantation

**Figure 1:**
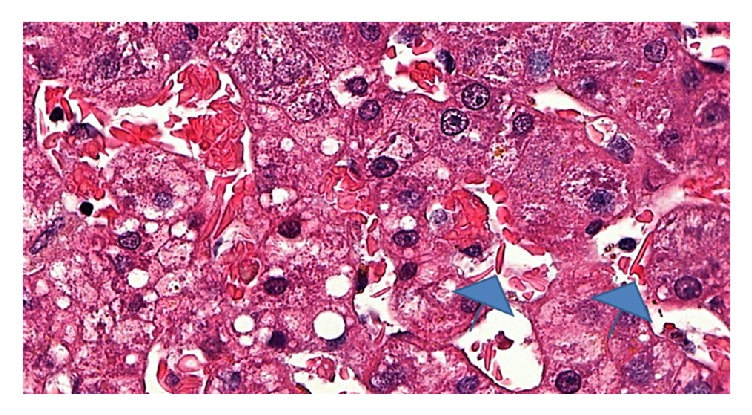
Sinusoidal distension and congestion with many crescent-shaped or needle-shaped red cells and sickle cells (arrowheads) are seen in this field, along with mild steatosis in hepatocytes (H&E, 500x, before reperfusion).

The liver recipient was a 37-year-old woman, and her blood type was the same as that of the donor. She weighed 54 kg and was 1.65 meters tall. Her BMI was 19.8 kg/m^2^. She was diagnosed with hepatitis B virus (HBV) cirrhosis and hepatocellular carcinoma (HCC) according to the Milan criteria (2 tumors each with diameter ≤ 3 cm, without extrahepatic and major vessel involvement). Model for end-stage liver disease (MELD) score was 18 and Child-Pugh-Turcotte (CPT) classification was B7. The serum alpha-fetoprotein (AFP) value was increasing recently to the transplant (>200 ng/ml). In the waiting list for liver transplant, the patient performed 3 transarterial chemoembolization (TACE) sessions, initially with completely treated areas but afterwards showing partial treatment (progression). The liver allograft weighed 1.495 kg. The cold ischemia time was 8 hours and 30 minutes, and the warm ischemia time was 38 minutes. The transplantation was performed on September 27th, 2016. The patient received four units of red blood cells, three units of platelets, and eight units of plasma during the surgery. The liver biopsy protocol included preclamping and prerevascularization biopsy with mild siderosis and steatosis, hepatocyte ballooning, rare canalicular cholestasis, and moderate sinusoidal congestion with predominance of drepanocytes (Figure 1).

**Figure 2:**
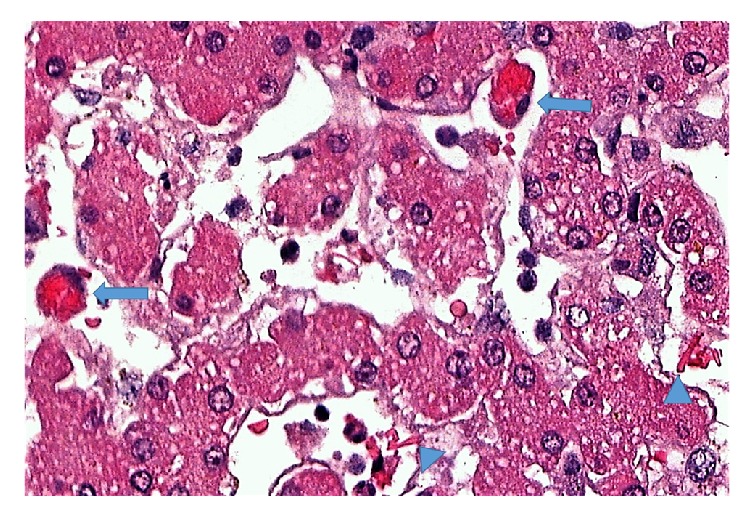
Kupffer cell erythrophagocytosis (arrows) and scarce sickle cells (arrowheads) in sinusoids, as well as some hepatocellular vacuolar degeneration (H&E, 500x).

The postrevascularization biopsy revealed mild ischemia reperfusion injury (grade 2), including apoptosis of hepatocytes and a minor neutrophilic sinusoidal infiltrate. Kupffer cell erythrophagocytosis and scarce sickle cells were still seen in a biopsy performed on the following day (Figure 2), along with some ischemic areas. The immunosuppression protocol included corticosteroids every day after the anesthetic induction for 6 months. In terms of the glucocorticoid withdrawal regimen, basiliximab 20 mg was administered on the anesthetic induction and on the fourth postoperative day; mycophenolate mofetil and tacrolimus were administered from the 4th day on (due to the institutional protocol and literature we initiated the onset of tacrolimus later due to acute renal failure and using protocol with corticosteroids and basiliximab). The tacrolimus dose (0.10-0.15 mg/kg/day administered twice a day) was adjusted based on the liver blood tests and the blood level 6-10 ng/ml was maintained during this first year.

### 2.3. Postoperative Course

The LT recipient also received antibiotics based on the medical profile of the liver donor: vancomycin and meropenem for seven days plus Amicacina for two days. Furthermore, she received hepatitis B immunoglobulin (HBIG) for seven days and then monthly for a year. She still receives entecavir (ETV) daily for preventing hepatitis B recurrence. As part of the LT protocol, she was submitted to a Doppler ultrasound of the vascular graft anastomosis investigation on the first postoperative day, and no abnormalities were noted. The follow-up of the vascular imaging examination will depend on clinical and laboratory data. Despite her good postoperative evolution, she developed acute kidney failure and she required hemodialysis for three weeks. The cause of acute renal failure and hemodialysis was due to liver transplantation procedure and the baseline renal function somewhat altered. Her recovery of renal function was complete, and she did not require renal replacement therapy anymore. She was discharged from hospital on the 37th postoperative day. Postoperative chemotherapy was not necessary due to good evolution and postoperative oncologic control image (CT) with absence of recurrence in the transplanted liver and decrease in the serum tumor marker (AFP <10 ng/ml). A long-term follow-up of 18 months revealed a good evolution, with normal liver blood tests.

### 2.4. Ethical Aspects

This study was approved by the Institutional Review Board and accomplished the entire requisite for studies in humans according to the guidelines of the 1975 Declaration of Helsinki.

## 3. Discussion

Sickle cell anemia (SCA) is a hereditary hemoglobinopathy and an atypical inherited disorder of hemoglobin called hemoglobin S (HbS). It is important due to its multifactorial components, diverse clinical manifestations, and impact on patient morbimortality [6]. Sickle cell disease (SCD) presents vascular manifestations leading to complications in multiple organ systems: vasoocclusion of hepatic sinusoids owing to the sickling way, hemosiderosis from chronic transfusions, viral hepatitis, and cholelithiasis in consequence of chronic hemolysis [6].

Some manifestations related to vascular damage have been associated with liver alterations due to acute hepatic crises to chronic liver disease accompanying intrahepatic cholestasis and iron overload. Regardless of this, intrahepatic cholestasis is possibly severe and clinical treatment is often inoperative [7]. SCA or SCD can offer higher morbid-mortality, more hospitalizations, and impaired quality of life [7]. In our case, we observed a nice macroscopic aspect of the liver in the donor harvesting and good donor laboratory tests and hemodynamic controls, so we decided to proceed with the LT. After that we confirm the good microscopic aspect of the liver with the preclamping graft biopsy.

Regarding SCA and SCD liver histology, as one of the limiting factors to use these patients as donors [7], the liver histological analysis is mandatory for a better interpretation of fibrosis, cholestasis, steatosis, and even cirrhosis. So, to maintain the donor's quality of the liver graft we recommend to perform a protocol histological analysis. That was carried out in our case and discarded any problem that reflects this good evolution.

There were few reported published cases of liver transplant in patients with SCD. Furthermore, a recent article review of the literature shows 10 adults and 5 children with SCD that underwent a liver transplant [7]. Orthotopic LT might be included as a treatment choice for SCD patients with end-stage liver disease and with chance to progress the disease with other treatments.

Elsiesy H et al. [8] in 2017 reported in a meeting abstract 7 cases with sickle cell trait patients for liver donation. They conclude that the liver donation is safe from sickle cell trait donors [8]. Nevertheless, we did not find any published articles focusing on donors with SCA.

We have highlighted a literature review and a successful LT case using SCA donor to a HBV cirrhosis and HCC patient with dropout risk from the waiting list for LT. The important objective of this work is to demonstrate this successful recipient and donor match that show that we can expand the donor criteria and share this successful option for liver transplantation. Furthermore, this present report is enhanced to promote to the transplant community this approach with this type of donor.

## 4. Conclusion

In summary, we have reported that a deceased liver donor with sickle cell anemia is an adequate option for LT. The articles that we found demonstrate the importance of using a safe donor and the real benefits of expanding use of organs.

## Abbreviations

LT: Liver transplantation
HBV: Hepatitis B virus
HCC: Hepatocellular carcinoma
BMI: Body mass index
SCA: Sickle cell anemia
SCD: Sickle cell disease
HbS: Hemoglobin, called hemoglobin S
MELD: Model for end-stage liver disease
CPT: Child-Pugh-Turcotte
HBIG: Hepatitis B immunoglobulin
ETV: Entecavir.
